# SIRT1 Serum Concentrations in Lipodystrophic Syndromes

**DOI:** 10.3390/ijms25094785

**Published:** 2024-04-27

**Authors:** Luisa Salvatori, Silvia Magno, Giovanni Ceccarini, Rossella Tozzi, Savina Contini, Caterina Pelosini, Ferruccio Santini, Lucio Gnessi, Stefania Mariani

**Affiliations:** 1 Institute of Molecular Biology and Pathology, National Research Council (CNR), c/o Sapienza University of Rome, 00185 Rome, Italy; luisa.salvatori@cnr.it; 2 Obesity and Lipodystrophy Center, Endocrinology Unit, University Hospital of Pisa, 56124 Pisa, Italygiovanni.ceccarini@unipi.it (G.C.);; 3 Department of Molecular Medicine, Sapienza University of Rome, 00161 Rome, Italy; 4 Department of Experimental Medicine, Section of Medical Physiopathology, Food Science and Endocrinology, Sapienza University of Rome, 00161 Rome, Italy; 5 Chemistry and Endocrinology Laboratory, University Hospital of Pisa, 56124 Pisa, Italy

**Keywords:** lipodystrophy, SIRT1, adipose tissue, adipokines

## Abstract

Lipodystrophies (LDs) are rare, complex disorders of the adipose tissue characterized by selective fat loss, altered adipokine profile and metabolic impairment. Sirtuins (SIRTs) are class III NAD^+^-dependent histone deacetylases linked to fat metabolism. SIRT1 plays a critical role in metabolic health by deacetylating target proteins in tissue types including liver, muscle, and adipose. Circulating SIRT1 levels have been found to be reduced in obesity and increased in anorexia nervosa and patients experiencing weight loss. We evaluated circulating SIRT1 levels in relation to fat levels in 32 lipodystrophic patients affected by congenital or acquired LDs compared to non-LD subjects (24 with anorexia nervosa, 22 normal weight, and 24 with obesity). SIRT1 serum levels were higher in LDs than normal weight subjects (mean ± SEM 4.18 ± 0.48 vs. 2.59 ± 0.20 ng/mL) and subjects with obesity (1.7 ± 0.39 ng/mL), whereas they were close to those measured in anorexia nervosa (3.44 ± 0.46 ng/mL). Our findings show that within the LD group, there was no relationship between SIRT1 levels and the amount of body fat. The mechanisms responsible for secretion and regulation of SIRT1 in LD deserve further investigation.

## 1. Introduction

Lipodystrophies (LDs), best defined as lipodystrophic syndromes, are a group of extremely rare and heterogeneous diseases characterized by selective loss of subcutaneous adipose tissue (SAT) in the absence of a hypercatabolic state or nutritional deprivation [1]. LDs display variable extent of fat loss with altered distribution of body fat [1,2]. Non-HIV LDs can be classified into four main categories: congenital generalized lipodystrophy (CGL), familial partial lipodystrophy (FPLD, predominantly type 1 and 2), acquired generalized lipodystrophy (AGL), and acquired partial lipodystrophy (APL). Additional subtypes include progeroid variants (PS) in which lipodystrophy is associated with premature aging [3]. Subcutaneous fat loss and decreased SAT plasticity are accompanied by an ectopic accumulation of fat leading to metabolic derangements such as insulin resistance, type 2 diabetes, dyslipidemia (mostly hypertriglyceridemia) and fatty liver disease [4]. With SAT being a dynamic hormone-secreting organ, LDs exhibit an altered adipokine profile. Indeed, leptin is extremely low in subjects with reduced amounts of SAT, such as patients with CGL and AGL [5,6], and severe hyperphagia occurs because of leptin deficiency. Serum concentrations of adiponectin are also reduced in LDs and correlate with the extent of fat loss [6,7].

Sirtuins (SIRTs) are class III NAD^+^-dependent histone deacetylases expressed in relation to fat level and energy variations linking protein acetylation to metabolism and response to oxidative stress [8]. In mammals, seven nonredundant SIRTs have been described: SIRT1, SIRT6, and SIRT7 are expressed in the nucleus, SIRT3, SIRT4, and SIRT5 are imported into mitochondria, and SIRT2 is cytoplasmic [9]. Their different cellular localizations allow the organisms to sense changes in energy status (assessed as NAD^+^/NADH levels) in all cellular compartments. Nuclear SIRTs deacetylate histones, thereby modulating gene expression based on cellular metabolic state through epigenetic mechanisms: the main targets are transcription factors and metabolic enzymes that program cells for oxidative metabolism in mitochondria [8]. In particular, SIRT1 plays a critical role in metabolic health by deacetylating target proteins in several tissue types, including liver, muscle, adipose, heart, and endothelium, and regulates effects on calorie restriction (CR), feeding behavior, body temperature, and energy expenditure [10,11,12]. During fasting or energy depletion SIRT1 expression and activity increase, thus promoting SAT lipolysis, increase in glycolytic and gluconeogenic pathways, and muscle and liver fatty acid oxidation. Some of the metabolic actions of SIRT1 are also mediated through the hypothalamus [13], and others by the adipose tissue. In fact, during fasting and CR, SIRT1 favors fat mobilization from adipose tissue to promote lipid oxidation in the liver and muscles [14]. Consistently, genetic ablation of SIRT1 in adipose tissue leads to increased adiposity and insulin resistance [15]. Conversely, excessive energy intake increases caspase 1 activity, thus causing cleavage of SIRT1 in adipose tissue [15].

The main source of serum SIRT1 and the mechanisms responsible for its regulation or secretion from its tissue origin are still unclear [16]. As other enzymes, SIRT1 is found in the blood, and measuring circulating SIRT1 could represent an indicator of health status. Accordingly, the decline in SIRT1 serum levels is a promising marker for early detection of endocrine and metabolic diseases [17], Alzheimer’s disease [18], cardiovascular disease [19], etc., and SIRT1 is reputedly a frailty-related biomarker [20]. Interestingly, serum SIRT1 levels were found to be reduced in obesity and increased in anorexia nervosa (AN) and in patients experiencing weight loss [21,22,23].

To further investigate the relationship between SIRT1 and variations in fat mass, in this study, we evaluated the association between SIRT1 serum concentrations and fat percentage in patients with different subtypes of LDs compared to non-LD subjects with different levels of fat deposits.

## 2. Results

We enrolled patients affected by LDs to be compared with patients with anorexia nervosa (AN), obesity and normal weight. The basic clinical characteristics of the groups are reported in Table 1.

Patients with LD had BMI and fat percentage comparable to those of normal weight subjects, whereas patients with anorexia and obesity had the lowest and highest BMI and fat percentage, respectively. The highest values of fasting plasma glucose (FPG) and triglycerides were observed in LDs.

**Figure 1:**
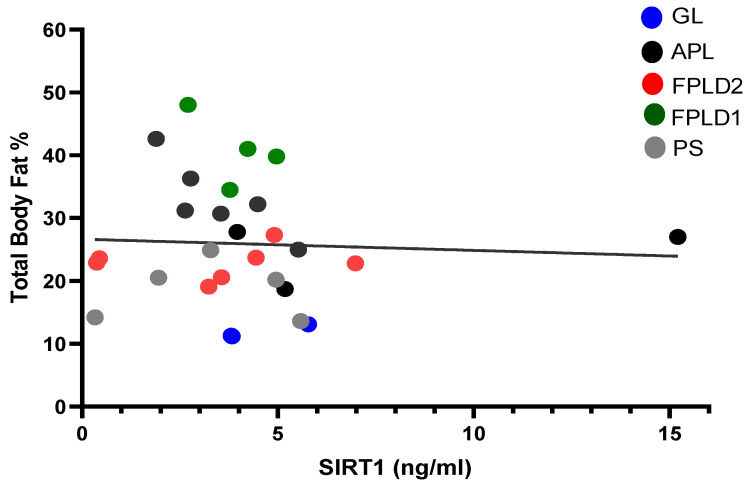
Correlation between fat percentage in the whole body and SIRT1 serum concentrations in patients with different subtypes of LD: generalized lipodystrophy (GL, n = 3), progeroid syndromes (PS, n = 5), familial partial lipodystrophy type 1 (FPLD1, n = 4), familial partial lipodystrophy type 2 (FPLD2, n = 7), and acquired partial lipodystrophy (APL, n = 10). r^2^ = 0.002489, *p* = 0.79.

As a starting point of our research, we were interested in understanding if, in the whole spectrum of lipodystrophic syndromes encompassing various amounts of adiposity, SIRT1 serum concentrations reflected the amount of fat tissue, expressed as percentage of fat over the total body mass. No relationship could be documented between fat percentage and SIRT1 concentrations (r = −0.152, *p* = 0.431), indicating that SIRT1 levels do not display significant differences attributable to varying amounts of fat in LDs (Figure 1). Furthermore, no correlation between SIRT1 and BMI, FPG, total and LDL cholesterol, or triglycerides was observed in LDs (Table S1 in the Supplementary Data).

**Figure 2:**
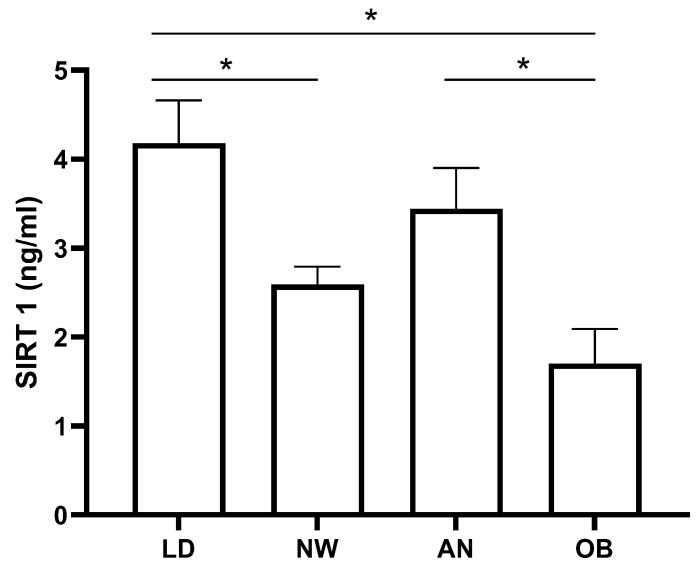
Comparison of SIRT1 serum concentrations in patients with lipodystrophies (LD, n = 32), anorexia nervosa (AN, n = 24), obesity (OB, n = 24) and normal weight subjects (NW, n = 22). Values are expressed as means ± standard error (SEM). * *p* < 0.05.

As expected, SIRT1 levels in the non-LD cohorts showed that obese patients’ levels were lower than those of the anorectic patients (*p* < 0.05) and normal weight subjects (*p* = 0.053) (Figure 2). The inverse relationship of SIRT1 with fat percentage in non-LD groups was demonstrated by the relative correlation analysis (r = −0.514, *p* < 0.0001).

Interestingly, SIRT1 levels in LDs were significantly higher than those measured in both normal weight and obesity (*p* < 0.05), while they were not different when compared to anorexia (*p* = 0.2) (Figure 2).

## 3. Discussion

SIRT1 activity is known to regulate fat metabolism, and several SIRT1 protein substrates are involved in maintaining homeostasis in adipogenesis. This prompted us to investigate SIRT1 serum levels in lipodystrophic patients.

LDs are fatty tissue disorders characterized by the absence, reduction, or abnormal distribution of adipose tissue. LDs are classified based on their etiology into congenital (and familial) or acquired and according to the distribution of fat loss into generalized or partial. Due to their rarity, LDs often pass unrecognized, and a significant diagnostic delay has been reported [24]. Appropriate LD management and treatment are essential to improve life expectancy and quality of life: diet and specific pharmacological interventions, as well as follow-up by a multidisciplinary team, are necessary [25]. Indeed, in LD patients, lack of SAT is associated with metabolic abnormalities such as insulin resistance and type 2 diabetes mellitus, liver steatosis, hypertriglyceridemia, increased risk of pancreatitis, and coronary heart disease [1,26].

In LDs, the inadequate subcutaneous fat storage is also associated with impaired adipokine secretion. The decrease in leptin and adiponectin has been extensively described in these disorders [7,27], and depending on fat loss, lower levels of adipokines were found in patients with generalized LDs (CGL and AGL) compared to partial FPLD and APL [6]. Contrastingly, in this study, we found that SIRT1 levels were substantially homogeneous regardless of the heterogeneity of fat levels in different LD subtypes, and thus no correlation with the amount of fat mass was evident.

Interestingly, LDs and obesity exhibit several commonalities, sharing ectopic fat deposition and overlapping clinical complications [28,29]. Low concentrations of SIRT1 have been found in obesity in both serum and tissue [21,30,31]. The reduction in SIRT1 is also evident in visceral adipose-derived stem cells of obese patients [32]. Intriguingly, although high levels of SIRT1 are generally indicative of good metabolic health [33], it must be noted that this does not apply to patients with LDs, who are subject to metabolic derangements and severe lipotoxicity [34].

We remark that our findings, instead of suggesting a relationship between metabolic derangements and SIRT1 levels, rather seem to point to a possible gradient of serum concentrations characterized by lower SIRT1 in obesity, intermediate in normal weight subjects, and higher in states of lower adiposity such as AN and LDs.

Studying circulating SIRT1 in healthy and diseased adipose tissue may have implications for a broader understanding of fat mass metabolism and metabolic disorders. Comprehending the pathophysiology underlying LD and its possible prognostic meaning leads us to continue investigating SIRT1 in lipodystrophic patients, also in light of the possible interactions of SIRT1 with the main adipogenic genes causing LD.

We have to acknowledge that our research has some limitations: (1) the low number of lipodystrophic subjects enrolled, due to the rarity of the disease, and the heterogeneity of LD patients; (2) the lack of in-depth metabolic characterization of our patient cohort; and (3) the limited knowledge on the possible relationships between serum SIRT1 levels and their tissue expression, activity, and origin.

In summary, serum SIRT1 levels were within a physiological range across all forms of LDs but did not reflect heterogeneous amounts of fat. The mechanisms responsible for secretion and regulation of SIRT1 from adipose tissue in LD need further studies, and disease-dedicated registries may offer this possibility.

## 4. Materials and Methods

### 4.1. Participants

The study was conducted in 32 patients with LD, 24 patients affected by AN, 22 normal weight healthy subjects and 24 patients with obesity.

The diagnosis of LD was made based on accepted criteria [25] and after exclusion of other causes of fat loss. Clinical data were obtained from the participants’ electronic medical records. Four patients were diagnosed with GL: three patients affected by Berardinelli–Seip syndrome confirmed by genetic testing documenting biallelic mutations of the 1-acylglycerol-3-phosphate O-acyltransferase 2 (*AGPAT2*) gene, and one patient affected by acquired generalized LD (Lawrence syndrome). Five patients were affected by PS: four with genetic testing positive for a heterozygous mutation of the lamin A/C (*LMNA*) gene, one with a p.R133L [35] and three with a p.R349W mutation, and the fifth patient with the heterozygous mutation p.R507C of the DNA polymerase delta 1 (*POLD1*) gene. Six patients were affected by FPLD1 (Köbberling syndrome) diagnosed based on accepted criteria [36] and after testing negative for mutations involved in genetic forms of LD. Seven patients were affected by FPLD2 (Dunnigan syndrome), with genetic testing confirming the pathogenic *LMNA* missense variant p.R482W/Q. Ten patients were affected by APL: six of them had Barraquer–Simons syndrome [37] and four had autoimmune-mediated subtypes of APL, one of whom underwent total body irradiation and hematopoietic stem cell transplant during childhood [38].

Patients affected by LDs were recruited at the Obesity and Lipodystrophy Center of the University Hospital of Pisa, Italy. Participants with AN, healthy normal weight and obesity were recruited at the Day Hospital of the Department of Experimental Medicine, Policlinico Umberto I, Sapienza University of Rome, Italy. The diagnosis of AN was made based on the *Diagnostic and Statistical Manual of Mental Disorders* (5th ed.; DSM-5 [39]) and after exclusion of other causes of fat loss [40].

The reduction in SAT and its altered distribution in patients with LDs, responsible for the onset and development of metabolic complications, led to disorders of varying severity requiring pharmacological intervention for most. Indeed, 65% of the LD patients (21/32) had diabetes or impaired glucose tolerance and used glucose-lowering agents (metformin, thiazolidinediones, glinides and/or liraglutide based on specific patient characteristics), and five of them were also on insulin treatment. Further, 81% of patients (26/32) had dyslipidemia, 15 of whom (58%) had isolated hypertriglyceridemia, 3 (11%) isolated hypercholesterolemia, and 8 (31%) combined hyperlipidemia. Dyslipidemic patients used specific lipid-lowering agents (statins and/or ezetimibe or cholestyramine; omega 3 polyunsaturated fatty acids and/or fibrates). Three of the LD patients were receiving pharmacological treatment with metreleptin. Hepatic steatosis was documented in 30 out of 32 patients (93%).

The study was conducted according to the Declaration of Helsinki, and all participants provided written informed consent for participation in the study and for the publication of their clinical and biochemical information.

### 4.2. Anthropometric Measurements

Height and body weight were measured by standard procedures to calculate BMI (kg/m^2^) as the ratio between weight (kg) and height squared (m^2^). Obesity was defined following WHO criteria when BMI was ≥30. Total and segmental body fat in the trunk and upper and lower extremities was evaluated by whole-body DEXA (Hologic, Discovery A, S/N 84551, Marlborough, MA, USA) in 29 lipodystrophic patients. The adiposity was expressed as the percentage of body fat mass over the total mass.

### 4.3. Biochemistry

All patients underwent venous sampling for the determination of FPG, total cholesterol, LDL cholesterol and triglycerides. Circulating SIRT1 was determined by a commercially available human ELISA kit (MyBioSource, San Diego, CA, USA) after 12 h fasting according to the manufacturer’s instructions.

### 4.4. Statistical Analysis

Data are expressed as means ± standard error (SEM). Simple linear regression analysis and unpaired *t*-tests were used. This study, assuming a minimum sample size of 22 patients in each group, had greater than 75% power to detect differences in SIRT1 serum concentration between groups. All the graphs, calculations, and statistical analyses were conducted using GraphPad Prism version 8.4.3. A two-sided *p* value of 0.05 was the criterion for statistical significance.

## 5. Conclusions

SIRT1 is a nuclear master regulator of energy homeostasis, and its concentrations measured in serum are reduced in obesity and increased in AN. SIRT1 serum levels in LDs did not reflect the amount of body fat and were higher than in normal weight subjects and obesity, and comparable to those measured in AN. Further investigation into the possible involvement of SIRT1 in the pathophysiology of LDs is valuable.

## Figures and Tables

**Table 1:** Characteristics of subjects with lipodystrophy (LD), anorexia nervosa, normal weight, and obesity.

	Lipodystrophy	Anorexia Nervosa	Normal Weight	Obesity
Subjects	32	24	22	24
Gender (male/female)	5/27	6/18	1/21	2/22
Age (years)	40.28 ± 3.94	27.96 ± 2.57	29.81 ± 1.87	35.71 ± 2.6
BMI (Kg/m^2^)	22.44 ± 0.95	15.43 ± 0.36	22.36 ± 0.27	37.77 ± 0.79
Total fat (%)	25.92 ± 1.7	16 ± 1.15	27.06 ± 1.45	41.33 ± 0.85
SIRT1 (ng/mL)	4.18 ± 0.48	3.44 ± 0.46	2.59 ± 0.2	1.7 ± 0.39
FPG (mg/dL)	99.06 ± 4.55	72.92 ± 1.76	82.45 ± 0.2	89.25 ± 1.62
Total cholesterol (mg/dL)	168.9 ± 5.53	178.08 ± 11.11	181.5 ± 6.66	168.41 ± 9.42
LDL cholesterol (mg/dL)	101.52 ± 6.95	88.17 ± 8.97	97.77 ± 5.32	100.74 ± 6.65
Triglycerides (mg/dL)	178.9 ± 26.29	95.5 ± 11.93	93.86 ± 12.47	104.63 ± 10.99

Values are expressed as means ± SEM. BMI, body mass index; FPG, fasting plasma glucose; LDL, low-density-lipoprotein cholesterol.

## Data Availability

Datasets generated and/or analyzed during this study are not publicly available, but are available from the corresponding author.

## Supplementary Materials

The following supporting information can be downloaded at https://www.mdpi.com/article/10.3390/ijms25094785/s1.
