# Supplementary material for: SIRT1 Serum Concentrations in Lipodystrophic Syndromes
Source: Int J Mol Sci. 2024 Apr 27;25(9):4785. doi: 10.3390/ijms25094785 (PMC11084952; doi:10.3390/ijms25094785)
Supplement: Supplementary file 1 [file ijms-25-04785-s001.zip › ijms-2935386-supplementary.pdf]

**Table S1. Correlation between SIRT1 and metabolic variables in LDs patients.**		
	**SIRT1**	
	**r**	**p-value**
**BMI (Kg/m^2)**	0.096	0.605
**Total Fat (%)**	-0.152	0.431
**FPG (mg/dL)**	-0.020	0.266
**Tot-Cholesterol (mg/dL)**	0.064	0.73
**LDL-Cholesterol (mg/dL)**	0.185	0.319
**Triglycerides (mg/dL)**	0.178	0.337

SIRT1 and metabolic indicators correlation in patients with lipodystrophy. SIRT1 was not correlated with any of the variables (p-value > 0.05). BMI, body mass index; FPG, fasting plasma glucose; LDL, low-density lipoprotein-cholesterol.
